# Supplementary material for: Developmental fronto-parietal shift of brain activation during mental arithmetic across the lifespan: A registered report protocol
Source: PLoS One. 2021 Aug 25;16(8):e0256232. doi: 10.1371/journal.pone.0256232 (PMC8386861; doi:10.1371/journal.pone.0256232)
Supplement: Attachment — Submitted filename: RR1_life_neuro_letter_1.pdf [file pone.0256232.s001.pdf]

Editor:

Dear Dr. Artemenko,

Thank you for submitting your registered report to PLOS ONE. I have sent it to two expert reviewers and have received their comments. As you will see at the bottom of this email, both reviewers found that the report was well motivated, well written and the proposed methodology sound and able to answer your hypotheses. I do agree with the reviewers on all of these points. Both reviewers, however, have a number of suggestions. I will not reiterate these here as the reviewers did a careful evaluation of your manuscript and the comments are quite clear. But I encourage you to take these into account in a revision of the report.

[…]

We look forward to receiving your revised manuscript.

Kind regards,
Jérôme Prado
Academic Editor
PLOS ONE

Dear Jérôme Prado,

Thank you for the possibility to resubmit my registered report protocol after revision. In the following, you can find my replies to the reviewers' comments and all marked changes in the revised manuscript.

I would like to thank the reviewers here in a short note: I was impressed by the quality, professionalism and kindness of the reviewers when evaluating my registered report. I never received so excellent reviews before and I highly appreciate it. I really think that all comments improved my manuscript in the revision and I am really curious about the review process at later stages (looking forward from stage I to stage II). Thank you!

Kind regards,
Christina Artemenko

Journal Requirements:

When submitting your revision, we need you to address these additional requirements.

1. Please ensure that your manuscript meets PLOS ONE's style requirements, including those for file naming. The PLOS ONE style templates can be found at https://journals.plos.org/plosone/s/file?id=wjVg/PLOSOne_formatting_sample_main_body.pdf and https://journals.plos.org/plosone/s/file?id=ba62/PLOSOne_formatting_sample_title_authors_affiliations.pdf

2. As this manuscript is a Registered Report Protocol, please ensure that you indicate this in your title.

3. In your Methods section, please state how you will obtain consent. Specifically, please state whether you will obtain consent from parents or guardians of the minors included in the study or whether the research ethics committee or IRB specifically will waive the need for their consent.

The revised manuscript follows the journal guidelines, includes "A Registered Report Protocol" in the title, and specified the procedures for informed consent in the Methods (cf. p. 10). Note that changes in the format were not marked.

Reviewer #1:

This study aims to investigate if developmental changes observed between childhood and young adulthood in the relative engagement of frontal and parietal regions during mental arithmetic would show continuity or change in older adulthood. Using a cross-sectional approach, the study will characterize the behavioral and neural effects during multi-digit mental arithmetic across different age groups. The lifespan approach is valuable, especially in light of the decline in general cognitive capacities in old age that may affect how mental arithmetic is accomplished in older adults to maintain the same level of performance. The focus on multi-digit mental arithmetic is practically motivated and a theoretically appropriate one given that it would tap heavily into the engagement of frontal regions implicated in working memory and executive functions. These cognitive constructs underlie the concept of the fronto-parietal shift. In general, the study is well motivated, and the protocol is sound and suitable for addressing the hypotheses. I am confident that the author will be able to execute the project successfully. The current version of the registered report is already very well written, but it would be great to have the author clarify and justify the hypotheses and predictions a little more. Below are my specific comments about these issues, as well other theoretical and methodological ones:

1. This first point is a general observation of how the literature has characterized the so-called fronto-parietal shift. Based on my own reading of the literature, the frontoparietal network is engaged during mental arithmetic even in adults (e.g., in an empircal study by Grotheer et al., 2019; or in a meta-analysis by Hawes et al., 2019). However, from the second paragraph on page 3, I am afraid that readers may get the impression that the putative shift is qualitative rather than quantitative. By qualitative, I mean there tends to be a simplified and inaccurate understanding that adults rely mainly on the parietal regions and less (or not at all) on frontal regions; and the reverse applies to children. By quantitative, I mean that whenever researchers present activation maps for children and adults separately, the fronto-parietal network is recruited regardless of age, but the "shift" emerges only when the maps are contrasted between age groups. I am not sure if the author shares the same sentiment, but it would be helpful if the author can be clearer about describing the so-called shift.

> I totally agree with the reviewer and understand the developmental fronto-parietal shift as a quantitative and not as a qualitative change in the activation of the frontal and parietal brain regions involved in arithmetic. I clarified the respective paragraph in the Introduction and also revised the hypotheses accordingly (cf. response to comment 6):
>
> "Arithmetic is represented in a fronto-parietal network in the brain (for meta-analyses in children and adults see Arsalidou et al., 2017; Arsalidou & Taylor, 2011; for a model and its extensions in adults see Dehaene et al., 2003; Klein et al., 2016; for a review in children see Peters & De Smedt, 2017), but the extent of frontal and parietal activation in this network changes during development. […] Altogether, there is evidence for a developmental shift from domain-general frontal activation towards more specific parietal activation within the fronto-parietal network of arithmetic (Menon, 2010; Rivera et al., 2005), when comparing children and adults." (cf. p. 4)

2. The author introduces task complexity as an additional layer of complication to the developmental fronto-parietal shift. It is definitely an interesting dimension, but it can be challenging to interpret the findings. As both the shift and task complexity implicate the frontal regions, the report at this stage will benefit from a consideration whether the effects are additive, or whether they interact and how they may interact. This will lead to more specific hypotheses, and also a better delineation between confirmatory and exploratory analyses. In the current version, the author does not indicate if any anticipated or post-hoc exploratory analyses will be performed.

> The fronto-parietal shift during development holds for arithmetic in general and will be replicated in a confirmatory analysis for the comparison of children and young adults and tested in an exploratory analysis for the comparison of young adults and the elderly. Developmental changes in the processing of arithmetic complexity and thus neural changes regarding the carry and borrow effects are less clear.

It might be that the developmental fronto-parietal shift is even more pronounced for complex arithmetic as compared to simple arithmetic (leading to similar assumptions for the developmental changes of the carry and borrow effects as for arithmetic in general), so that the neural carry and borrow effects decrease during development (interaction of age and complexity in addition to main effects). However, this is not necessarily the case (for a detailed discussion of this issue see my response to comment 8 and the respective changes in the manuscript, cf. p. 7). Therefore, planned exploratory analyses will be conducted concerning the developmental changes of the neural carry and borrow effects in the frontal brain areas.

3. Please clarify and elaborate on what is meant by "continuous processing characteristics of the carry effect" on page 4. Although the author alluded to a distinction between "categorical" (i.e., with and without carry or borrow operations) and "continuous", it is not immediately clear whether "continuous" (as assessed based on unit sum) refers to within each condition, just within the condition with carry/borrow operations, or across conditions (i.e., collapsed across all trials). Readers who are not familiar with previous work (and would not have read the methods section) may not be familiar with the distinction and why they matter. It would therefore be necessary to elaborate on these types of processing characteristics by providing more concrete examples.

The description of the continuous processing characteristics of the carry and borrow operations was extended by definitions and examples:

"Continuous processing characteristics of the carry operation are indicated by the unit sum (e.g., 58 + 36 has a unit sum of 8 + 6 = 14), which theoretically ranges from 0 to 18 (carry operation necessary when unit sum ≥ 10); continuous processing characteristics of the borrow operation are indicated by the unit difference (e.g., 94 – 36 has a unit difference of 4 – 6 = –2), which theoretically ranges from –9 to +9 (borrow operation necessary when unit difference < 0)." (cf. p. 5 f.)

4. In several paragraphs on page 4 and beyond, I believe that "carry/borrow effects" might have been mistakenly used in place of "carry/borrow operations". For instance, on page 4, "rely on their initial place-value understanding for addition performance and the carry effect later in elementary school". An "effect" as described earlier in the introduction refers to the difference in performance between two conditions (with and without carry/borrow operations). It is also not immediate clear to me the distinction between "place-value understanding" and carry operation in this context. The clarity of the paper can be enhanced if the author could check the specificity and intended contextualized meanings of these terms. The author may also choose to provide their own nuanced definitions.

I agree that the use of the correct terminology is of high importance. In the revised manuscript, I introduced the levels of place-value processing and used the terms afterwards consistently. I further revised the manuscript regarding the use of "carry/borrow operations" and "carry/borrow effects". If there is any instance of incorrect terminology left, I will be happy to correct it.

Introduction of the levels of place-value processing: "Knowledge of the place-value system is crucial for multi-digit number processing and consists of three levels: place identification, place-value activation, and place-value computation (Nuerk et al., 2015). Multi-digit arithmetic is especially difficult when it requires operations across place-values, i.e., place-value computation (Nuerk et al., 2015). This is the case for the carry operation in addition […] and the borrow operation in subtraction […]." (cf. p. 5)

Differentiation between categorical and continuous effects regarding place-value processing: "While the categorical carry and borrow operations require place-value computation – the highest level of place-value processing – the continuous carry and borrow operations instead require place-value activation (Nuerk et al., 2015)." (cf. p. 5)

Revised sentence mentioned above: „Place identification, the first level of place-value processing, serves as a precursor for arithmetic performance and place-value computation later in elementary school (Moeller, Pixner, et al., 2011).“ (cf. p. 6)

5. The author noted that frontal involvement found in the carry and borrow effects reflects "domain-general demands like working memory for task difficulty due to the categorical effects" (p. 5). However, frontal involvement may not always reflect domain-general demands (e.g., see Fedorenko & Blank, 2020 for prefrontal involvement specific to language or multiple demands; see Sokolowski et al., 2017 for frontal involvement across many basic numerical tasks). The association between frontal involvement and domain-general demands is a common reverse inference, but does not appear to have strong empirical evidence for. Would the author consider including an independent working memory task (or a relevant task) to directly test for the hypothesis of working memory/domain-general demands so as to strengthen the inferences that could be made from this study? This may also help clarify whether parietal activation truly reflects "domain-specific magnitude processing associated with the continuous effects" (p. 5), or can be partly accounted for by working memory (because unit sums can also involve procedural computation rather than fact retrieval).

In agreement with the reviewer, the congruence of domain-general vs. domain-specific processes and their representation in the frontal vs. parietal cortex is not exclusively given. To reflect this issue, all instances in the Introduction are relativized by quantifiers (e.g., "mostly", p. 7). Nevertheless, the distinction between domain-general vs. domain-specific processes in the context of arithmetic seems helpful when assuming that domain-specific relates to number magnitude processing in particular (and not generally to arithmetic processing) and domain-general relates to executive functions such as working memory. Based on the meta-analysis of Sokolowski et al. (2017), number magnitude processing indeed involves not only parietal brain regions, but also the superior frontal gyrus. However, the current study focuses on the inferior and middle frontal gyri (instead of the superior frontal gyrus). Due to the differentiation between a task-based and an effect-based approach that also addresses categorical and continuous processing characteristics, the underlying mechanisms of the carry and borrow effects might be further classified when identifying different neural activation patterns for categorical and continuous effects (in this case, the effects might represent localizers for different processes involved in arithmetic, at least to a certain extent).

As noted by Fedorenko & Blank (2020) the inferior frontal gyrus plays a role in domain-general processes, but also in the left-hemispheric language network (specific for processing language and not number magnitudes). Including a localizer task, unfortunately, would only partly solve the issue. On the one hand, it is an adequate method to localize different functions (e.g., working memory, number magnitude processing), and to use these locations as ROIs in the analysis on arithmetic. On the other hand, the spatial resolution of fNIRS might be not enough to disentangle these areas, e.g., domain-general processing areas vs. language-specific areas in the left IFG (as identified in Fedorenko & Blank, 2020). Thus, inferences based on localizer tasks might even be wrong (e.g., the working memory task activates a certain channel, effects of arithmetic will be found for this channel, but the inference that domain-general processes might subserve arithmetic in this case might nevertheless be wrong, since the channel might capture activation from adjacent areas as well).

Instead, I would like to address this issue as a limitation of the current study (in the future Discussion of the paper) and suggest future research with localizer tasks by using fMRI as an appropriate technique. The interpretation of the activation differences found will be based on previous studies, will carefully comprise alternative explanations regarding domain-general and domain-specific processes, and will be related to the behavioral effects in arithmetic performance and possible differences in other measures such as working memory (in this regard, exploratory analyses on interindividual differences regarding these measures can be considered).

6. For H1, please clarify and justify the hypothesis that "children should mainly rely on frontal regions (left IFG and bilateral MFG), whereas elderly should mainly rely on parietal regions (left IPS) in comparison to

young adults" (p. 5). In particular, what does "mainly rely on" means. It can be interpreted as "only involves". Alternatively, the author may mean "should recruit to a greater extent frontal/parietal regions". Also, why would that be the case for the elderly? Is it possible that there is no quantitative difference between the elderly and young adults? Could the elderly instead recruit frontal regions to a greater extent than young adults (i.e., children-like), independent of what is observed for the parietal activation?

To clarify first: The expression "mainly rely on" should be understood in terms of "should recruit to a greater extent", reflecting a quantitative and not a qualitative change in brain activation. The hypothesis was rephrased accordingly.

Because of the missing evidence for age-related neural changes in the elderly, several outcomes seem possible (more/less/similar frontal/parietal activation). The continuation of the fronto-parietal shift is one possible outcome, stability, decline or compensation are other possible outcomes. This means that the research question is exploratory and therefore will be treated as exploratory in the hypotheses.

The hypothesis was reformulated in order to reflect the exploratory character of this research question: "Moreover, the neural correlates of arithmetic will be explored in the elderly: if the developmental fronto-parietal shift generalizes to the whole lifespan, the elderly might show less frontal activation (left IFG and bilateral MFG) and increased parietal activation (left IPS) in comparison to young adults; however, if arithmetic is not affected by aging or is affected and needs compensation, the elderly might show similar or increased frontal activation and similar or less parietal activation in comparison to young adults." (cf. p. 8)

7. For H2, could the behavioral carry and borrow effects be equivalent between young adults and elderly as well? This alternative hypothesis seems plausible given that previous research found that "carry and borrow operations are not impaired and might be even superior in older as compared to younger adults, as reflected by similar or smaller carry and borrow effects" (p. 4).

It is correct that previous studies show the carry/borrow effects to be larger or similar – but not smaller – in young adults as compared to the elderly. Therefore, the hypothesis was rephrased accordingly:

"Regarding the lifespan development, the behavioral carry and borrow effects are expected to be larger in children than in young adults and larger or similar in young adults as compared to the elderly, replicating previous research." (cf. p. 8)

8. For H2, the author also hypothesized that "neural development of effects… might decrease during the lifespan due to the fronto-parietal activation shift for arithmetic in general" (p. 5). It is not immediately clear what this means, and it is also made complicated by the conflation of task-based and effect-based neural effects. Are these neural effects additive, or do they interact? It would be good to unpack this more carefully so that the planned analyses can be better evaluated. To address this, the author should provide clearer hypotheses about how the extent of the frontal and parietal activations may manifest in the age-by-condition (with and without carry/borrow operations) interaction in relation to the fronto-parietal shift framework. Finally, related to the other issues brought up, it is not clear what the author means by "when considering interindividual performance differences where smaller behavioral carry and borrow effects were associated with larger neural effects for frontal activation due to math ability, the neural carry and borrow effects might also increase during lifespan" (p. 6). It is not apparent why individual differences are considered here when the primary questions are about group averages. Also, why would smaller behavioral carry and borrow effects be associated with larger frontal activation? It would help with understanding the logic if the author could unpack these parts a little more.

The hypothesis on the developmental changes of the neural carry and borrow effects in the frontal brain regions is exploratory and two outcomes can be assumed based on the literature: either the fronto-parietal shift can be applied to a larger extent to complex arithmetic than to arithmetic in general so that due to automatization of the carry and borrow operations during the lifespan frontal activation associated

with these operations decreases and thus the neural carry and borrow effects decrease during development; or the neural effects change in dependency with the corresponding behavioral effects, when considering that performance concerning the carry and borrow operations increases during the lifespan resulting in smaller behavioral effects and thus larger neural effects, as it was shown in a study on interindividual differences (Artemenko et al., 2018). In this study, high-skilled individuals efficiently used the frontal resources for the carry and borrow operations (larger neural effect due to additional frontal activation for carry/borrow problems as compared to non-carry/borrow problems, smaller behavioral effect), whereas low-skilled individuals needed these resources even for problems without carry or borrow operation (smaller neural effect due to high frontal activation for both problems with and without carry/borrow operation, larger behavioral effect).

In order to introduce the developmental changes, some sentences on these considerations have been added: "Due to the developmental fronto-parietal shift in brain activation for arithmetic in general, the neural activation might also change for processing arithmetic complexity. For instance, frontal activation associated with the carry and borrow operations might decrease during lifespan development due to automatization, since the use of domain-general processes becomes more efficient from childhood to adulthood, and the general cognitive decline restricts further efficient use of domain-general processes during aging. On the other hand, a study on interindividual differences in math ability showed that smaller behavioral carry and borrow effects might be associated with larger neural effects (in high- as compared to low-skilled individuals), since high-skilled individuals efficiently used the frontal resources for the carry and borrow operations whereas low-skilled individuals needed these resources even for problems without carry or borrow operation (Artemenko, Soltanlou, et al., 2018). Thus, a decrease of the behavioral carry and borrow effects during lifespan development – with individuals getting better in the carry and borrow operations – might be associated with an increase in the neural carry and borrow effects in frontal brain regions. The current study aims to investigate the behavioral and neural correlates of the carry and borrow effects in children, young adults, and in the elderly, and to explore developmental changes." (cf. p. 7)

Consequently, the part of the hypothesis was rephrased: "The neural development of the effects will be explored here for the first time: the neural carry and borrow effects might either decrease during the lifespan, reflecting the fronto-parietal activation shift particularly for complex arithmetic, or increase during the lifespan, reflecting the more efficient use of frontal resources with increasing performance." (cf. p. 8 f.)

9. For H3, it is not immediately clear based on the discussion in the introduction why the carry and borrow effects will no longer be categorical in elderly. This is especially so based the point raised in Comment #7. Is there a prediction for the development of parietal activation for the continuous carry and borrow effects?

The hypothesis based on the developmental fronto-parietal shift assumes that the importance of domain-general and domain-specific processes for arithmetic changes: children rely more on domain-general processes than adults and adults rely more on domain-specific processes than children. When considering that the categorical carry and borrow operations mostly are subserved by domain-general processes and the continuous carry and borrow operations mostly by domain-specific processes, the developmental findings are line with the shift hypothesis: children show mainly categorical processing characteristics, and young adults show additional continuous processing characteristics (in addition to the categorical processes) and express smaller carry and borrow effects. Unfortunately, there is no data for the processing characteristics in the elderly yet. Since the domain-general cognitive resources decline during aging, it might be assumed that the elderly mainly rely on domain-specific resources – and thus mainly show continuous processing characteristics –, because they still show comparable or even superior performance for the carry and borrow operations as compared to young adults (regardless of the general cognitive decline, which might be compensated with preserved or even more automatized

number magnitude processing). In the context of categorical and continuous effects, the statement about the elderly was added in the Introduction:

"Considering the general cognitive decline during aging, the carry and borrow effects might be mainly driven by domain-specific processes in the elderly, but empirical evidence is still missing." (cf. p. 6)

On a neural level, the categorical carry effect is associated with frontal activation (reflecting domain-general processes, working memory, task difficulty), while the continuous carry effect is associated with parietal activation (reflecting domain-specific processes, number magnitude processing, problem size), as described in the Introduction. H3 states that a parametric investigation of unit sum/difference is considered to be associated with parietal activation in each age group (cf. p. 9). The question on developmental effects in this context is interesting, however, there is no clear hypothesis. It could be speculated that if the carry and borrow effects are not driven by continuous processing characteristics in children (evidence for null effects on the behavioral level), parietal activation can also not be observed in relation to unit sum and difference. This might lead to higher parietal activation in the direct comparison of young adults to children. It might also be assumed that if the elderly mainly rely on continuous processing characteristics and are more automatized in number magnitude processing, parietal activation might be higher when compared to young adults, indicating further functional specialization. However, these speculations do not inspire theory driven hypotheses and thus developmental differences in parietal activation due to unit sum and difference might only be considered in additional exploratory analyses depending on the behavioral and neuroimaging results for the confirmatory analyses. (This is different from the planned exploratory analysis for the developmental changes of the categorical effects, because exploratory hypotheses can be stated based on behavioral developmental findings in case of the categorical effects, but there is no such behavioral data for the continuous effects yet.)

10. For clarity, please justify why 3rd and 4th grades will be used. Is it because multi-digit arithmetic instruction only starts in 2nd grade?

In grade 1, children learn to calculate in the number range up to 20. In grade 2, children learn to calculate up to 100 and learn the carry and borrow operations. In grades 3 and 4, children are supposed to know how to add and subtract two-digit numbers and also how to execute carry and borrow operations. Therefore, these two grades (second half of elementary school) are the target in this study which aims at investigating children that just recently learned the carry and borrow operations and now have this knowledge. In a behavioral longitudinal study investigating the borrow effect in subtraction in children from grade 3 to 4 (Artemenko, Pixner, Moeller, & Nuerk, 2018), we demonstrated that it is possible to assess two-digit arithmetic even in grade 3, however, we needed to exclude a considerable number of children due to a high error rate so that the investigation of children in grade 2 does not seem feasible for the current study. This justifies the target grades 3 and 4, *but not younger*.

The following information was added to the revised manuscript: "Children learn the carry and borrow operations for single-digit and two-digit arithmetic in the first two years of elementary school and afterwards are able to use these skills (cf. Artemenko, Pixner, et al., 2018)." (cf. p. 6)

Besides knowledge and feasibility, also the underlying mechanisms of arithmetic indicate that changes regarding the fronto-parietal shift in brain activation (e.g., Rivera et al., 2015) and regarding the decrease of carry and borrow effects (e.g., Lemaire & Callies, 2009) can rather be detected after elementary school, while changes during elementary school are less clear (conflicting findings for neural activation: Rosenberg-Lee et al., 2011; Qin et al., 2014; no significant changes in the borrow effect: Artemenko, Pixner, Moeller, & Nuerk, 2018). This justifies the target grades 3 and 4, *but not older*.

In the objectives, the explanation of the different age groups was added: "The study will target crucial stages of development: elementary school children in grades 3 and 4, because they just acquired the skills for two-digit arithmetic and thus serve as a starting point of lifespan development, young adults,

because they have already established their arithmetic skills and thus serve as a reference for development, and the elderly, because they are experienced in using their arithmetic skills but might show a general cognitive decline and thus serve as a final point of lifespan development." (cf. p. 7 f.)

11. The use of sequential Bayes factor design used to estimate sample size is commendable and well-described. Please provide citation(s) for "80% of studies with an infinite sequential sampling stop earlier than nmax. If sampling is terminated because of reaching nmax, only with a probability of 5% the study will obtain misleading evidence", or whether a simulation was conducted.

A simulation was conducted to estimate these properties of the design. This information was added to the Methods introducing the values:

"The properties of the planned research design were estimated with Monte Carlo simulations according to Schönbrodt and Wagenmakers (2018): […]" (cf. p. 17).

In case all the information on the simulation are not necessary to be stated in the registered report protocol, I would also agree to reduce the chapter to the only relevant information on the sample size estimation and effects of interest.

12. The arithmetic task involves both addition and subtraction that will be analyzed as separate levels of the operation factor. However, H1 – H3 mainly focus on age, complexity, and age-by-complexity interaction. Hence, at present, the hypotheses (nothing about operation per se) and analyses (operation as a factor of interest) are not congruent. Please reconcile these differences as appropriately guided by theory (e.g., provide hypotheses about three-way interactions, focus on one operation, or collapse across operations for the primary planned analyses, but analyze them separately for planned exploratory analyses, etc.).

The processes underlying the carry operation in addition and the borrow operation in subtraction are considered to be similar. Most behavioral and neuroimaging studies focus on addition and it is often assumed that findings from research in addition can be generalized to subtraction, however, literature on subtraction is quite sparse. This motivates the use of both arithmetic operations in the current study.

Nevertheless, subtraction is more difficult than addition and the borrow effect might be larger than the carry effect. This is why arithmetic operation will be considered as a factor in the analyses in case operation-specific effects will be found (with follow-up exploratory analyses). However, there is no evidence for operation-specific effects concerning lifespan development. Therefore, no developmental hypotheses can be derived from the literature and all hypotheses are formulated for both arithmetic operations in parallel.

For transparency, I added these sentences after the stated hypotheses: "The carry and borrow effects will be investigated concerning arithmetic complexity in two-digit addition and subtraction, respectively. The processes underlying these arithmetic operations are similar, while subtraction is more difficult than addition (Artemenko, Soltanlou, et al., 2018). However, there is not yet evidence for operation-specific differences in relation to lifespan development." (cf. p. 9)

13. For measuring reaction time of mental arithmetic using button press, how will the author account for instances in which the button presses are accompanied by irrelevant verbalizations (e.g., "erm") or followed by self-corrections? How reliable is this procedure compared to audio recording of verbal responses and manually extracting reaction times thereafter?

The use of voice keys or audio recordings to extract reaction times might have some advantages, but also faces several problems concerning parameter choices (e.g., thresholds differ between the numbers words and certain letters, manual extraction might be imprecise and based on several subjective choices, less automatization in data collection leads to less transparency of the Open Data published afterwards) and practical issues (e.g., ethical considerations for audio recordings, disturbed recordings due to pandemic measures such as face masks). Therefore, the originally planned measures for reaction times by button press will be used in the current study but improved to ensure data quality.

Three measures will be taken to ensure the adequate recording of reaction times: (1) An intensive practice phase will precede the arithmetic task in order to familiarize the subjects with the response format and allow for corrections of the procedures by the experimenter. In case of problems, the practice phase can be repeated. (2) As soon as a button will be pressed, the arithmetic problem will disappear on the screen to enforce the subjects to calculate before (and not during) button press and oral response. (3) The subjects will be instructed to keep the button pressed until finishing their response and the button release will be recorded in addition to button press. Button press will be used as dependent variable; however, trials will be excluded from analysis if the duration between button press and button release deviates more than 3 median absolute deviations from the subject's median. This procedure should ensure to exclude trials including irrelevant verbalizations of self-corrections.

In the description of the arithmetic task details were added as following: "In an oral production paradigm, the subjects will be asked to mentally solve the problem as quickly and accurately as possible and to respond while pressing the space bar. Button press and button release will be recorded and the experimenter will blindly note the given responses. Each stimulus will be presented with a time limit of 30 s and disappear upon button press to emphasize mental arithmetic before responding." (cf. p. 12)

In the description of the procedures details were added as following: "The practice phase of the arithmetic task will consist of 12 trials to familiarize the subjects with the response format (which can be repeated)." (cf. p. 13)

In the description of the analysis details were added as following: "Trials will be excluded from the RT analysis […] when the duration between button press and button release deviates more than 3 median absolute deviations from the subject's median in the arithmetic task […]." (cf. p. 15)

14. For outlier detection, the author may want to consider using the median absolute deviation instead of standard deviation based on the mean. See Leys, C., Ley, C., Klein, O., Bernard, P., & Licata, L. (2013). Detecting outliers: Do not use standard deviation around the mean, use absolute deviation around the median. Journal of Experimental Social Psychology, 49(4), 764–766. https://doi.org/10.1016/j.jesp.2013.03.013.

The argument of Leys et al. (2013) is convincing so that the RT data analysis will exclude participants and trials based on the median absolute deviation.

The revised outlier detection for subjects reads as following: "Moreover, an outlier analysis will be conducted specific to the respective age group to exclude subjects deviating more than 3 median absolute deviations from the group's median in RT of the arithmetic task from all analysis (cf. Leys et al., 2013)." (cf. p. 15)

The revised outlier detection for trials reads as following: "Trials will be excluded from the RT analysis (arithmetic and working memory task) as well as from the fNIRS analysis (arithmetic task) […] when the RT deviates more than 3 median absolute deviations from the subject's median in the respective task (cf. Leys et al., 2013)." (cf. p. 15)

15. Why is SMG a recorded channel, but not a region of interest? If it is used for exploratory analyses, please describe what the analyses may be.

The role of the SMG in arithmetic is not as clear as for the other regions of interest so that no clear hypothesis can be stated. However, this region cannot be neglected since age-related effects were sometimes found for the SMG as well (e.g., Rivera et al., 2005). Therefore, the SMG was listed as a region of interest as well, but will not be included in the confirmatory analyses but rather in exploratory analyses, i.e., conducting the main analysis with brain activation in the SMG as a dependent variable. (The same holds for the AG.) I added it to the revised manuscript in the analysis section after the description of the main analysis:

"Planned exploratory analyses will be conducted on ER and beta values for neural activation in the other ROIs (left/right AG, SMG)." (cf. p. 17)

16. It appears that BF > 3 is used as a cutoff for inferences, but the Bayes factor design analysis used BF = 6 as a criterion. Please reconcile this difference. Please also provide the scale values for all default priors used, e.g., the width of the Cauchy prior for t-tests (0.707 is the default in JASP).

The use of the criterion of $BF \geq 6$ is now the same for the Bayes factor design analysis (to collect data until enough evidence will be obtained) and the planned Bayesian posthoc *t*-tests (for effects in the Bayesian ANOVAs). The stopping rules for data collection are irrelevant for data interpretation. Therefore, the interpretation of *BF*s (with moderate evidence between 3 and 10) according to Jeffreys (1961) and Lee & Wagenmakers (2013) remains unchanged.

Bayes factor design analysis: "In this design, data collection (1) will start with a minimum sample size of $n_{min} = 20$ per group, (2) will continue until a BF_{10} or $BF_{01} \geq 6$ is obtained for all effects of interest, or (3) will be stopped when a maximum sample size of $n_{max} = 60$ per group has been reached." (cf. p. 17)

Planned Bayesian posthoc *t*-tests: "For effects with BF_{10} or $BF_{01} \geq 6$, post-hoc two-sided tests will be conducted for the contrast children vs. young adults and the contrast young adults vs. the elderly by means of Bayesian independent samples *t*-tests, or for contrasting different conditions by means of Bayesian paired *t*-tests." (cf. p. 17)

The values for the priors were added as well: "The analysis prior will be set to a Cauchy prior scale of 0.707 in Bayesian *t*-tests, reflecting that H_0 and H_1 are equally likely to occur, to the default Cauchy prior of $r = 0.5$ for the fixed effects in Bayesian ANOVAs, to the default Jeffreys–Zellner–Siow prior of $r = 0.354$ for regression coefficients, and to a uniform model prior in Bayesian linear regressions." (cf. p. 16)

17. The author may want to consider using mixed models instead of repeated-measures ANOVA and simple regression to better handle missing data or even use trial level data instead of averaging across trials. Bayesian mixed models can be conducted in JASP. This is just a suggestion, and the author's choice will not affect my recommendation in any way.

I welcome this suggestion, because linear mixed models would be able to reflect both participant level as well as trial level data and I applied such analyses in previous studies. Bayesian linear mixed models, on the other hand, are rather advanced analyses: After reading and without experience I am not able to exactly plan such an analysis before having access to the data, which would be necessary for the registered report protocol. Therefore, I would like to stick to the planned Bayesian ANOVAs and regressions for the planned confirmatory data analyses. The data will be freely accessible as Open Data after publication so that further analyses might be conducted in terms of linear mixed models by myself or other researchers.

18. In the final paragraph of "Statistical data analysis", ER was not mentioned as a dependent variable in all the effects of interest, contrary to the last sentence of the preceding paragraph. Please reconcile this difference.

The hypotheses and corresponding confirmatory behavioral analyses will focus on RT as the important dependent variable in the arithmetic task. Additional exploratory analyses on ER will only be conducted for completeness and the results will be later reported in the Supplementary Material. I clarified the distinction between confirmatory and exploratory analyses in the revised manuscript:

"All confirmatory analyses will be conducted on the dependent variables RT, indicating behavioral performance, and beta values, indicating neural activation for each ROI separately (left/right IFG, MFG, IPS, AG). Planned exploratory analyses will be conducted on ER and beta values for neural activation in the other ROIs (left/right AG, SMG)." (cf. p. 17)

19. Will other cognitive measures, such as processing speed, working memory, etc., be included as covariates in the primary analyses, especially if they differ between the age groups?

The main focus of this study is to investigate age-related differences in arithmetic, first of all regardless of other age-related differences in cognition. Therefore, the main analyses will not consider additional cognitive measures as covariates, as age differences necessarily imply these cognitive differences and a correction would bias the results regarding arithmetic.[1] What might be interesting nevertheless, would be to further uncover the mechanisms of age-related differences in arithmetic considering these cognitive measures. Therefore, relevant cognitive measures should be considered in exploratory analyses aiming at the mechanisms underlying the age-related differences in arithmetic.

Minor Comments

1. In the abstract, "borrow" might be missing from "carry and operations".

The missing word was added (cf. p. 3).

2. The full labels associated with some acronyms are not provided, e.g., SPL and IPL in the second paragraph of page 3.

The abbreviations are introduced in the revised manuscript (cf. p. 4).

3. The timeline may be a little optimistic given that a maximum of 60 subjects per group is expected to be collected. I suggest allocating more time for data collection.

Data collection is now planned for a duration of a year from June 2021 (starting as soon as possiblt) until May 2022 (cf. p. 18).

References

Fedorenko, E., & Blank, I. A. (2020). Broca's Area Is Not a Natural Kind. Trends in Cognitive Sciences, 1–15. https://doi.org/10.1016/j.tics.2020.01.001

Grotheer, M., Zhen, Z., Lerma-Usabiaga, G., & Grill-Spector, K. (2019). Separate lanes for adding and reading in the white matter highways of the human brain. Nature Communications, 10(1), 3675. https://doi.org/10.1038/s41467-019-11424-1

Hawes, Z., Sokolowski, H. M., Ononye, C. B., & Ansari, D. (2019). Neural underpinnings of numerical and spatial cognition: An fMRI meta-analysis of brain regions associated with symbolic number, arithmetic, and mental rotation. Neuroscience and Biobehavioral Reviews, 103(May), 316–336. https://doi.org/10.1016/j.neubiorev.2019.05.007

Sokolowski, H. M., Fias, W., Mousa, A., & Ansari, D. (2017). Common and distinct brain regions in both parietal and frontal cortex support symbolic and nonsymbolic number processing in humans: A functional neuroimaging meta-analysis. NeuroImage, 146(October 2016), 376–394. https://doi.org/10.1016/j.neuroimage.2016.10.028

[1] To illustrate what I mean: Imagine that you meet a child, a younger adult and an older adult in daily life. You ask them to mentally calculate a complex arithmetic problem. First, I would like to know how the processes subserving their mental arithmetic differ, just because they differ in age. Because they differ in age, they probably have different cognitive resources. Thus, they might use different cognitive resources for mental arithmetic. This by itself is interesting. What would happen, if I would immediately correct for their different cognitive resources, i.e., align the cognitive resources (by e.g., increasing working memory for the older adult, decreasing processing speed for the younger adult, etc.)? I would observe age differences or similarities in arithmetic that might not reflect the reality. Therefore, this will be a second step aiming at the mechanisms behind the age differences.

Reviewer #2:

The protocol looks rigorous and I think that it has potential to contribute to the literature. I don't see any major issues with this registered report. I only have several minors issues that might need to be clarified:

1. The introduction is very well written, but I found it difficult to understand exactly what was the difference between categorical and continuous borrows/carries. I am not sure whether this just refers to a methodological way of categorizing the stimuli (i.e. two binary groups vs. taking into account more stimulus properties) and/or correspond to measurable cognitive processes. For instance, the author refers to studies showing that the effect of borrow/carry is categorical in elementary school. Could we conclude that the borrow/carry effect is so strong within young children that it "hides" effects other stimulus properties? By extension, could we conclude that this carry/borrow effect would decrease with age, so that other effects (such as the unit sum) are "visible"? I would advise the author to clarify this distinction, since it is crucial for their later analyses. Optionally, the author might want to provide some examples or a categorical vs. continuous carry/borrow processes.

(1) Categorical means that the decision whether or not a carry/borrow operation is necessary divides arithmetic problems into two categories (with or without carry/borrow operation). Continuous means that the unit sum/difference (on which the decision is based on) characterizes arithmetic problems based on the continuous values of the sum/difference of the units of the operands in an arithmetic problem (i.e., parametric based on stimulus properties). The description of the continuous processing characteristics of the carry and borrow operations was extended by definitions and examples:

"Continuous processing characteristics of the carry operation are indicated by the unit sum (e.g., 58 + 36 has a unit sum of 8 + 6 = 14), which theoretically ranges from 0 to 18 (carry operation necessary when unit sum ≥ 10); continuous processing characteristics of the borrow operation are indicated by the unit difference (e.g., 94 – 36 has a unit difference of 4 – 6 = –2), which theoretically ranges from –9 to +9 (borrow operation necessary when unit difference < 0)." (cf. p. 5 f.)

(2) Furthermore, the cognitive and neural processes differ: The categorical and continuous carry and borrow effects concern different levels of place-value processing, i.e., place-value computation and activation, respectively: "While the categorical carry and borrow effects reflect place-value computation – the highest level of place-value processing – the continuous carry and borrow effects instead reflect place-value activation (Nuerk et al., 2015)." (cf. p. 5)

The categorical carry effect is associated with frontal activation (reflecting mostly domain-general processes, working memory, task difficulty), while the continuous carry effect is associated with parietal activation (reflecting mostly domain-specific processes, number magnitude processing, problem size) (cf. p. 7).

(3) The hypothesis based on the developmental fronto-parietal shift assumes that the importance of domain-general and domain-specific processes for arithmetic changes: children rely more on domain-general processes than adults and adults rely more on domain-specific processes than children. When considering that the categorical carry and borrow operations mostly are subserved by domain-general processes and the continuous carry and borrow operations mostly by domain-specific processes, the developmental findings are line with the shift hypothesis: Children show mainly categorical processing characteristics, because the decision and execution of the carry or borrow operation are paramount for them, since they just learned these operations. Young adults, on the other hand, are proficient in using these operations – thus show smaller carry and borrow effects – and number magnitude processing is more automatized, leading to additional continuous processing characteristics (in addition to the categorical processes). Finally, there is no data for the processing characteristics in the elderly yet, but it might be assumed that they show mainly continuous processing characteristics, since the domain-general resources decline during aging but they still show comparable or even superior performance for the carry and borrow operations. Taken together, both categorical and continuous processes are involved

in the processing of the carry and borrow operations, however, during development one or the other might be more dominant due to the change in domain-general and domain-specific resources for arithmetic. In the revised Introduction, the argument for the developmental changes of the underlying processes of the carry and borrow effects was extended:

“Furthermore, the underlying processing characteristics for these effects might change, since the carry and borrow effects seem to be categorical effects in elementary school children (Artemenko, Pixner, et al., 2018), while the carry effect in young adults relies on continuous processing characteristics as well, as assessed by the unit sum (Klein, Moeller, et al., 2010; Klein, Willmes, et al., 2010; Moeller, Klein, et al., 2011). This suggests that primarily domain-general processes are driving the carry and borrow effects in children, while in young adults, domain-specific processes are additionally driving the carry effect. Considering the general cognitive decline during aging, the carry and borrow effects might be mainly driven by domain-specific processes in the elderly, but empirical evidence is still missing.” (cf. p. 6)

2. Relative to H1, "whereas the elderly should mainly rely on parietal regions (left IPS) in comparison to young adults." It seems to me that the author considers that the shift "continues" until old age. I am not sure that there are experimental evidence for this. It could alternatively that the shift is mature at adulthood and stay stable across life. That being said, it might be that elderly show less frontal activation (relative to young adults) due to general decline in frontal executive processes. Could the author specify/justify whether they expect old participants to rely less on frontal region due to the shift or to general cognitive decline in executive functions?

Because of the missing evidence for age-related neural changes in the elderly, several outcomes seem possible (more/less/similar frontal/parietal activation). The continuation of the fronto-parietal shift is one possible outcome, stability, decline or compensation are other possible outcomes. This means that the research question is exploratory and therefore will be treated as exploratory in the hypotheses.

The question whether less frontal activation during aging would reflect the fronto-parietal shift OR the general cognitive decline needs to be reconsidered (I will argument that it is not an “either – or”). In my opinion, the developmental fronto-parietal shift is a theoretical construct (stating originally that children show frontal activation to a lesser extent than adults) and the mechanisms behind the frontal activation decrease are interesting by themselves (e.g., the higher frontal engagement in children reflects domain-general processes, because they just learn arithmetic, use these general resources to manage these complex arithmetic tasks and will later automatize processes). A possible frontal activation decrease during aging might provide evidence for a continuation of the shift. The interpretation of this frontal activation decrease, however, might reflect the general cognitive decline during aging so that the general cognitive decline might be reason why the activation shift generalizes to development during adulthood. In sum, it is not a question either developmental shift or general cognitive decline but rather two questions addressing the shift on a theoretical level and the general cognitive decline in terms of possible mechanisms. (The same holds for the parietal activation increase.)

A sentence was added to the paragraph on aging in the Introduction: “The investigation of the mechanisms underlying arithmetic in the elderly brain will provide insights into whether the developmental fronto-parietal shift continues during aging, and whether arithmetic is preserved during aging or shows deficits due to the general cognitive decline, thus requiring compensation.” (cf. p. 5)

The hypothesis was reformulated in order to reflect the exploratory character of this research question: “Moreover, the neural correlates of arithmetic will be explored in the elderly: if the developmental fronto-parietal shift generalizes to the whole lifespan, the elderly might show less frontal activation (left IFG and bilateral MFG) and increased parietal activation (left IPS) in comparison to young adults; however, if arithmetic is not affected by aging or is affected and needs compensation, the elderly might

show similar or increased frontal activation and similar or less parietal activation in comparison to young adults." (cf. p. 8)

3. The author state that data collection "(2) will continue until a BF10 or BF01 of 6 is obtained". There are three main hypotheses, with several sub-hypotheses. Which hypothesis/es would be the criterion for this BF value?

Data collection will continue until BF ≥ 6 for all effects of interest according to the hypotheses. The structure of the Methods was changed so that after the analysis section the section on the Bayes factor design analysis is placed, which includes sample size estimation and the effects of interest:

Sample size estimation: "In this design, data collection (1) will start with a minimum sample size of n_{min} = 20 per group, (2) will continue until a BF_{10} or $BF_{01} \geq 6$ is obtained for all effects of interest, or (3) will be stopped when a maximum sample size of n_{max} = 60 per group has been reached." (cf. p. 17)

Effects of interest: "The effects of interest according to the hypotheses include the main effect of age (ANOVAs) for RT and activation in left IFG, bilateral MFG, and left IPS according to H1; the main effect of complexity and the interaction effect of complexity and age (ANOVAs) for RT and activation in left IFG and bilateral MFG according to H2; the categorical carry/borrow effect and the continuous effect of unit sum/difference in each age group (regressions) in RT and neural activation in left IPS according to H3." (cf. p. 18)

4. Could you specify what is the maximum number of channels to be interpolated for each individual?

I suggest a maximum of 3 interpolated channels (out of 18 channels) per subject. I revised the exclusion criteria for neural data analysis accordingly:

"Subjects will be excluded only from neural data analysis in case of more than 50% missing neural data of the arithmetic task (due to drop out, experimental or technical problems, trial exclusion and artifact rejection), or in case of more than 3 noisy channels (restricting channel interpolation to a maximum of 3 channels)." (cf. p. 15)

5. Very very minor: please provide the full name of SPL and IPL acronyms in the introduction

Right, the abbreviations are introduced in the revised manuscript (cf. p. 4).
